# Regional and patient-related factors influencing the willingness to use general practitioners as coordinators of the treatment in northern Germany - results of a cross-sectional observational study

**DOI:** 10.1186/s12875-020-01180-3

**Published:** 2020-06-17

**Authors:** Heike Hansen, Ingmar Schäfer, Sarah Porzelt, Agata Kazek, Dagmar Lühmann, Martin Scherer

**Affiliations:** Department of Primary Medical Care, University Medical Center Hamburg-Eppendorf, Martinistr, 52 20246 Hamburg, Germany

**Keywords:** General practice, General practitioner-centred healthcare, Healthcare utilisation, Regional comparison, Urban-rural differences

## Abstract

**Background:**

In most countries, the general practitioner (GP) is the first point of contact in the healthcare system and coordinator of healthcare. However, in Germany it is possible to consult an outpatient specialist even without referral. Coordination by a GP might thus reduce health expenditures and inequalities in the healthcare system. The study describes the patients’ willingness/commitment to use the GP as coordinator of healthcare and identifies regional and patient-related factors associated with the aforementioned commitment to the GP.

**Methods:**

Cross-sectional observational study using a standardised telephone patient survey in northern Germany. All counties and independent cities within a radius of 120 km around Hamburg were divided into three regional categories (urban areas, environs, rural areas) and stratified proportionally to the population size. Patients who had consulted the GP within the previous three months, and had been patients of the practice for at least three years were randomly selected from medical records of primary care practices in these districts and recruited for the study. Multivariate linear regression models adjusted for random effects at the level of federal states, administrative districts and practices were used as statistical analysis methods.

**Results:**

Eight hundred eleven patients (25.1%) from 186 practices and 34 administrative districts were interviewed. The patient commitment to a GP attained an average of 20 out of 24 possible points. Significant differences were found by sex (male vs. female: + 1.14 points, *p* < 0.001), morbidity (+ 0.10 per disease, *p* = 0.043), education (high vs. low: − 1.74, *p* < 0.001), logarithmised household net adjusted disposable income (− 0.93 per step on the logarithmic scale, *p* = 0.004), regional category (urban areas: − 0.85, *p* = 0.022; environs: − 0.80, *p* = 0.045) and healthcare utilisation (each GP contact: + 0.30, *p* < 0.001; each contact to a medical specialist: − 0.75, *p* = 0.018). Professional situation and age were not significantly associated with the GP commitment.

**Conclusion:**

On average, the patients’ commitment to their GP was relatively strong, but there were large differences between patient groups. An increase in the patient commitment to the GP could be achieved through better patient information and targeted interventions, e.g. to women or patients from regions of higher urban density.

**Trial registration:**

The study was registered in ClinicalTrials.gov (NCT02558322).

## Background

In most countries, healthcare is organised as a primary care system which usually means the general practitioner is also the first medical contact point in the healthcare system coordinating patients’ healthcare across sectors [1–3]. This role of general medicine is also referred to as gatekeeping. Studies indicate that a well-functioning GP coordination might reduce, among others, healthcare spending and inequalities in the healthcare system [4–7].

In Germany, however, free choice of physician, ie, the possibility to consult a medical specialist without previous GP consultation, is upheld as a fundamental principle of medical care. Even so, the general practitioner is often the first contact point for a sick person and the specialist group of general practitioners is among the most frequently consulted groups of physicians in Germany [1, 8].

The so-called “commitment to a general practitioner” is a concept, investigating to what extent patients voluntarily use their GP’s gatekeeping role or whether they move independently in the healthcare system instead. According to our definition, a strong commitment to a GP exists if 1) patients have a GP whom they prefer to consult first in all healthcare issues, 2) if patients understand their GP to be a central and competent coordinator of all their healthcare issues and 3) if there is a relationship of trust between patients and GP.

Regarding commitment to a GP, there seem to be vast differences between groups of patients and healthcare regions. This can be illustrated by models of the so-called “GP-centred healthcare” (HzV), which were introduced as special tariffs in Germany’s statutory health insurances in 2004. By enrolling in HzV, the participants commit themselves to consult a medical specialist only after referral by a contracted general practitioner [9]. Participation is voluntary for insured persons and GPs. The health insurance companies can offer the insured person advantages, eg, reduced co-payments in the pharmacies, but the HzV is not related to a different insurance premium than the normal tariff. There is a higher ratio of patients participating in HzV in less favoured rural areas. City dwellers, however, participate less frequently in HzV [10, 11]. Aside from the influence of the region where the patients live, participation in HzV is also associated with patient factors, eg, advanced age or existing chronic medical conditions [12].

However, it can be assumed that the commitment to a GP is probably not identical to the willingness to use HzV. A major role whether patients with a strong commitment to a GP use HzV might play, for example, the tariff fixing by health insurances, GP’s attitude towards HzV, patients’ data protection needs and psychological factors, such as individualism or general scepticism towards participating in interventions. The question, too, to what extent the long-time GP offers HzV or whether participating in HzV will only be possible by changing the GP, might influence participating in GP models independent of the commitment to a GP.

So far, the commitment to a GP as an independent concept has scarcely been investigated. The study presented here thus aims at describing the study population’s commitment to a GP with an in-house developed measuring tool and investigating to what extent the residential region, patient factors, and use of GPs and medical specialists are associated with the commitment to a GP.

## Methods

The study presented here is based on the cross-sectional observational study “Outpatient Healthcare Research North (*Ambulante Versorgungsforschung Nord – AVFN*)”. The methods of this study had been entered in the study register ClinicalTrials.gov (NCT02558322) before starting the survey and described in the published study protocol [13]. Although our study has been based on GP and patient interviews, the analysis presented here includes patient data only. The results from the GP interviews are published elsewhere [14].

### Study regions and regional categories

Three categories had been defined for the regional comparison based on the so-called “structural settlement of district types” of the German Federal Institute for Research on Building, Urban Affairs and Spatial Development [15]. The category “rural areas” included sparsely populated rural districts, the category “environs” urbanised districts and rural districts with signs of agglomeration, and the category “urban areas” independent large cities constituting districts in their own right.

For determining the survey area pursuant to the approach described in the study protocol [13], all administrative districts (counties and independent cities) were included in the study where at least 20% of the land area was located within a radius of 120 km (approx. 75 miles) linear distance around the study centre (University Medical Center Hamburg-Eppendorf). The thus chosen administrative districts for the study were derived from the German Federal States of Bremen, Hamburg, Mecklenburg-Western Pomerania, Lower Saxony, Saxony-Anhalt and Schleswig-Holstein. The specific districts and cities are shown in detail in the Tables A-C in Additional file 1.

### Recruitment of study participants

The study participants’ recruitment was carried out in two stages. At first, GPs were identified who had been accredited as statutory health insurance physicians in the respective administrative districts. This was achieved by using the database of the Department of Primary Medical Care at the University Medical Center Hamburg-Eppendorf as well as the databases of the respective regional associations of statutory health insurance physicians.

For the selection of GPs, a quota sampling design was chosen in order to be able to provide a representative picture of all regionally different healthcare situations in the study. The purpose of this design was to raise the probability of also including underserved regions into the study where usually many GPs were unwilling to participate in a study due to their heavy workload. The goal of the study was to recruit at least 80 GPs per regional category. The sample was stratified into individual administrative districts and the sample size in each district was fixed proportionally to the respective population size.

Subsequently, GPs thus identified were contacted in writing and invited to participate in the study. GPs were only eligible to participate in the study if they used an EDP system facilitating drawing up a list of all patients treated over the preceding quarter (3-month accounting period).

In stage two, every participating GP practice created a complete list from their electronic patient files of all patients who had been at least 18 years of age, had consulted the GP within the previous 3 months, and had been patients of the respective practice for a minimum of 3 years. Patients were randomly selected from this list and reviewed by the GP relating to the exclusion criteria until 15 eligible patients for the study had been identified and could be invited in writing to participate in the study. Patients were excluded if they had no capacity to give informed consent (eg, dementia), lacked German language skills and/or had functional limitations prohibiting a survey by telephone.

All participating patients signed a declaration of informed consent after they had received written and verbal information by their GP. The study was approved by the Ethics Commission of the Hamburg Medical Association on 12 August 2013 (file number PV 4535).

### Data collection and patient questionnaire

The data was collected during the time frame of 13 July 2015 to 25 April 2017. The participating patients were interviewed by telephone using a standardised questionnaire. The questionnaire contained, among others, questions regarding sociodemographic factors, education and household income, state of health, frequency of contacts with GPs and medical specialists as well as commitment to the GP.

Education and vocational qualification were classified pursuant to the international CASMIN classification into three groups [16]: 1) low, ie, inadequately completed general elementary education or basic vocational qualification, 2) medium, ie, intermediate qualification or general maturity certificate, and 3) high, ie, lower or higher tertiary education. In order to facilitate a comparison between the study participants, the household income was equivalence-weighted, ie, divided by the number of household members by weighting the head of the household with “1”, additional adult household members with “0.5” and children and young adults below the age of 15 years with “0.3”. The natural logarithm of the equivalised disposable income was calculated for the statistical inference analyses as a non-linear connection was assumed with the commitment to the GP.

Commitment to the GP was collected by using the questionnaire on intensity of the commitment to the GP (*“Fragebogen zur Intensität der Hausarztbindung (F-HaBi)”*). F-HaBi is a patient questionnaire examining the attitudes and behaviour regarding utilisation of GPs and medical specialists. It was developed at the Department of Primary Medical Care at the University Medical Center Hamburg-Eppendorf. The questionnaire is made up of six statements with the patient responding to each using the 5-step Likert scale as to what degree he agrees or disagrees. The patient’s answers are combined into a total score between 0 to 24 points. Higher scores of the total score of commitment to the GP indicate that the patient more likely recognises and uses the GP as coordinator. Lower scores indicate that the patient prefers to move independently in the healthcare system. The F-HaBi questionnaire is shown in the Additional files 2 (original German version) and 3 (English translation).

### Statistical analyses

In the first step, data analysis was carried out using descriptive statistics. Chi-squared-tests and t-tests were carried out to analyse the differences of commitment to a GP between the regions and to describe the differences in sociodemographic data and the healthcare utilisation between patients with a strong and low commitment to their GP. In the process of analysing the difference regarding the commitment to the GP, the F-HaBi total score was dichotomised. Low commitment to the GP was assigned if the F-HaBi score was below the median and strong commitment to the GP was assigned if the score was equal to or above the median.

The correlation between residential region, patient-related influencing factors, utilisation of GPs and medical specialists, and commitment to GPs was analysed using multivariate linear regression models adjusted for nested random effects at the levels of federal states, administrative districts and GP practices. The potential predictors of commitment to GPs were thus gradually included in three models. A possible improvement of the model fit by including additional variables compared to the next variable-reduced nested model was determined by the likelihood ratio test.

A psychometric validation was carried out to determine the suitability of the F-HaBi questionnaire. At first, the internal consistency of the construct with Cronbach’s α was determined where α ≥ 0.6 was defined to be the threshold score for sufficient consistency. The one-dimensional property of the construct was then tested with an exploratory factor analysis. Factors with an eigenvalue of ≥1.0 were extracted. Items were assigned to a dimension (= a factor) if the factor loading was ≥0.3. In a final step, the item-total correlation was determined with Pearson correlations between item scores and the Part-Whole-corrected summary score. The threshold score r ≥ 0.3 was defined to be a satisfying item-total correlation.

Data processing and data analysis were carried out using Stata 15.1. An alpha level of 5% (*p* ≤ 0.05) was defined to be statistically significant for all analyses of inferential statistics.

## Results

### Patient recruitment

With the included GP practices, 34 of the 37 selected administrative districts (91.9%) could be displayed in the data set. A map of the respective regions can be found in Schäfer et al. 2020 [14]. Only three districts of the environs (Delmenhorst, Diepholz and Osterholz) could not be included into the study. At first, 280 GPs were recruited from the selected regions. However, no patients could be recruited from 65 GPs due to time-related or organisational reasons (eg, sick primary care partners, problems with the patient management software). In the end, the patients of a total of 215 GPs were selected and contacted.

**Fig. 1:**
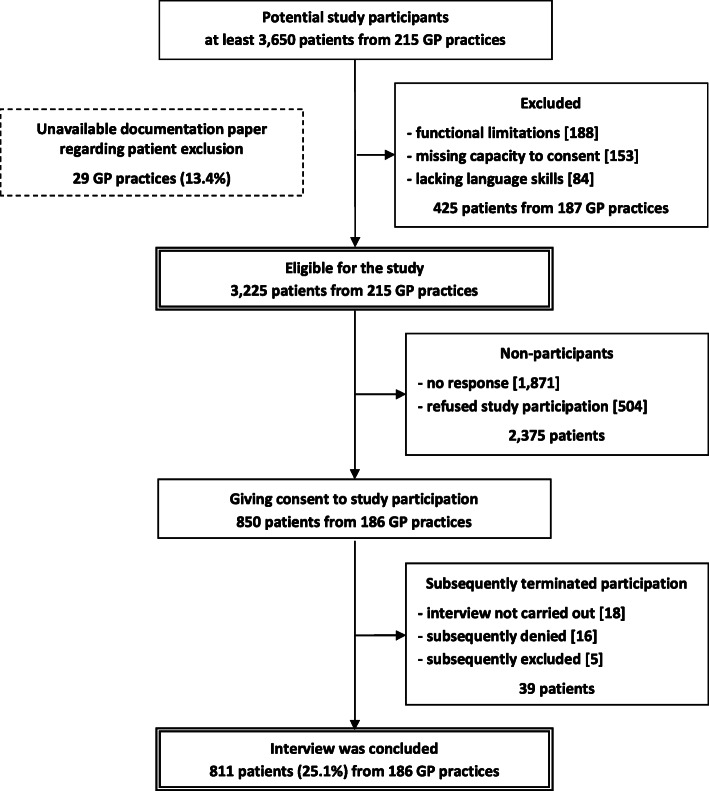
Recruitment process in the patient population

Figure 1 shows the patients’ recruitment process. During patient recruitment, each participating GP checked his patient list regarding exclusion criteria and excluded non-eligible patients. However, patient exclusion could not be documented in a structured way in 29 of the 215 practices due to the GPs’ sometimes very heavy workload. In the remaining 187 practices, the GPs documented that 188 patients (5.8%) were excluded due to functional limitations, 153 (4.7%) due to missing capacity to consent and 84 (2.6%) due to lacking language skills.

Altogether, 3225 patients from 215 practices were contacted by mail. Of those, there was no response from 1871 (58.1%) patients, 504 (15.7%) refused to participate, and 850 patients agreed to participate in the study. In the end, a total of 39 patients had to be excluded because the interview could not be carried out for different reasons (18 patients), the participation in the study had been withdrawn (16 patients) or it became clear during the telephone call that an exclusion criterion applied retrospectively (5 patients). In total, 811 patients (25.1%) from 186 GP practices could be interviewed. Each of the aforementioned 34 administrative districts was represented by at least one participating GP practice and at least one interviewed patient. Tables A to C of the Additional file 1 describe the recruitment process of the stratification groups.

### Patient characteristics and the degree of commitment to the GP

The sociodemographic data of the participating patients are listed in Table 1. On average, study participants had been 63 years of age and 58% of them female. Most of the patients had been from the region “rural areas”. The majority of the population had general or intermediate qualifications and more than half of the study participants had already entered retirement. The average equivalised disposable income was approximately 1800 Euro per month. On average, patients suffered from five chronic medical conditions, had 1.9 times personal contact with their GP, and had consulted 0.6 times a medical specialist over the previous 3 months. High blood pressure, chronic back/neck pain and osteoarthritis were the most common medical conditions mentioned by patients (s. Table 2).

**Table 1:** Patient population according to degree of commitment to GP

	In total (*n* = 807)	Low commitment to GP (*n* = 394)	Strong commitment to GP (*n* = 413)	p
***Age (in years)***	***62.8 ± 13.8***	***60.9 ± 14.5***	***64.7 ± 12.9***	***< 0.001***
***Sex***				***0.001***
***female***	***57.7%***	***63.7%***	***52.1%***	
***male***	***42.3%***	***36.3%***	***47.9%***	
Regional category				0.176
urban areas	28.1%	31.0%	25.4%	
environs	33.3%	33.0%	33.7%	
rural areas	38.5%	36.0%	40.9%	
***Education pursuant to CASMIN***				***< 0.001***
***low***	***40.3%***	***32.0%***	***48.2%***	
***medium***	***43.5%***	***45.0%***	***42.1%***	
***high***	***16.2%*** (*n* = 802)	***23.0%*** (*n* = 391)	***9.7%*** (*n* = 411)	
Professional situation				
***retiree/pensioner***	***54.5%***	***47.7%***	***61.0%***	***< 0.001***
***employed***	***30.3%***	***36.4%***	***24.3%***	***< 0.001***
self-employed/freelancer	5.7%	7.2%	4.2%	0.072
housewife/homemaker	3.2%	2.8%	3.5%	0.599
***job-seeking/unemployed***	***2.8%*** (*n* = 793)	***1.5%*** (*n* = 390)	***4.0%*** (*n* = 403)	***0.037***
***Equivalised disposable income****(in Euro per month)*	***1795 ± 877*** (n = 786)	***1928 ± 951*** (*n* = 385)	***1667 ± 779*** (*n* = 401)	***< 0.001***
***Number of chronic medical conditions****(relating to 39 categories)*	***4.9 ± 3.3***	***4.6 ± 3.3***	***5.3 ± 3.4***	***< 0.001***
***Number of contacts with GP****(last 3 months)*	***1.9 ± 2.0*** (*n* = 794)	***1.6 ± 1.4*** (*n* = 389)	***2.3 ± 1.5*** (*n* = 786)	***< 0.001***
***Number of contacts with medical specialists****(last 3 months)*	***0.60 ± 0.49*** (*n* = 806)	***0.64 ± 0.48*** (n = 394)	***0.56 ± 0.50*** (*n* = 412)	***< 0.019***

Statistically significant results (*p* ≤ 0.05) are shown in bolt and italic; Low commitment to GP = below the median; Strong commitment to GP = equal to or above the median

**Table 2:** Patient self-reported chronic conditions according to degree of commitment to GP

	In total (*n* = 807)	Low commitment to GP (*n* = 394)	Strong commitment to GP (*n* = 413)	p
***High blood pressure***	***52.4%***	***47.0%***	***57.6%***	***0.002***
Chronic back pain/neck pain	44.7%	41.6%	47.7%	0.083
Osteoarthritis/mechanical arthritis	42.1%	39.6%	44.6%	0.154
Cutaneous nerve dysfunction	27.0%	28.7%	25.4%	0.298
High blood lipid levels	24.8%	22.6%	26.9%	0.158
Chronic sleep problems	24.8%	23.6%	25.9%	0.449
Dizziness/vertigo	23.2%	20.6%	25.7%	0.086
Thyroid disorders	22.8%	24.6%	21.2%	0.229
Chronic gastritis/gastro oesophageal reflux	18.1%	16.8%	19.4%	0.334
Severe visual problems	16.7%	16.2%	17.2%	0.718
Urinary incontinence/bladder weakness	16.2%	16.2%	16.2%	0.994
Heart arrhythmias	15.6%	14.7%	16.5%	0.495
***Diabetes mellitus***	***13.6%***	***10.2%***	***17.0%***	***0.005***
Depression	11.8%	10.9%	12.6%	0.460
***Chronic fatigue***	***9.9%***	***7.6%***	***12.1%***	***0.033***
Migraine/chronic headache	9.9%	11.4%	8.5%	0.161
Chronic skin conditions	9.7%	11.2%	8.2%	0.158
***Heart diseases/CHD***	***9.5%***	***5.3%***	***13.6%***	***< 0.001***
Somatoform disorders	9.3%	8.6%	9.9%	0.526
Enlarged prostate	8.4%	7.6%	9.2%	0.417
Anxiety disorders	7.9%	7.1%	8.7%	0.397
Asthma	7.8%	6.9%	8.7%	0.324
Malignant tumours	7.7%	6.6%	8.7%	0.259
Gynaecological problems	7.3%	7.6%	7.0%	0.747
***Atherosclerosis/PAOD***	***7.1%***	***4.6%***	***9.4%***	***0.007***
Increased susceptibility to infections	7.1%	5.3%	8.7%	0.061
Heart failure	6.7%	5.3%	8.0%	0.131
COPD/chronic bronchitis	6.4%	6.4%	6.5%	0.911
Rheumatoid joints/soft tissue rheumatism	6.3%	5.8%	6.8%	0.582
Elevated uric acid level/gout	6.0%	5.6%	6.3%	0.669
Osteoporosis	6.0%	4.3%	7.5%	0.055
Intestinal wall hernias/diverticula	5.7%	5.8%	5.6%	0.869
Status post stroke	4.2%	3.6%	4.8%	0.362
Liver diseases	4.0%	3.6%	4.4%	0.558
***Cardiac valve disorders***	***3.8%***	***2.3%***	***5.3%***	***0.025***
Renal failure/weak kidney function	3.5%	3.6%	3.4%	0.899
Gallstones/gallbladder inflammation	3.2%	3.1%	3.4%	0.782
Lack of red blood cells/anaemia	2.5%	2.5%	2.4%	0.915
Kidney stones/concrements in the ureter	1.9%	1.5%	2.2%	0.490

Statistically significant results (p ≤ 0.05) are shown in bolt and italic; Low commitment to GP = below the median; Strong commitment to GP = equal to or above the median

Table 3 shows the study participants’ answers on the questionnaire regarding the degree of their commitment to their GP (F-HaBi). Between 81 and 95% of the patients agreed with “fully agree” or “mostly agree” to the first four more action-related statements concerning their GP commitment and 72% stated that they trusted their GP more than any other physician. 94% of them rated the GP information on their treatment as very good. Rural study participants stated more often than urban study participants to consult their GP in advance and get referrals from their GP before seeing a medical specialist.

**Table 3:** Patient self-report on commitment to GP from F-HaBi

	Total	Urban areas	Environs	Rural areas	p (u/r)	p (e/r)
When I have health problems, I visit my GP first.					0.290	0.625
fully agree	70.4%	66.2%	69.1%	74.5%		
mostly agree	24.7%	28.5%	26.0%	20.7%		
not sure	1.0%	1.3%	0.7%	1.0%		
mostly disagree	3.0%	2.6%	3.4%	2.9%		
fully disagree	1.0% (*n* = 811)	1.3% (*n* = 228)	0.7% (*n* = 269)	1.0% (*n* = 314)		
***When I think that I have to see a medical specialist, I consult my GP in advance.***					***0.011***	0.121
***fully agree***	66.0%	***57.7%***	65.8%	***72.2%***		
***mostly agree***	19.9%	***25.6%***	17.1%	***18.2%***		
***not sure***	3.3%	***3.5%***	4.1%	***2.6%***		
***mostly disagree***	5.4%	***6.6%***	6.7%	***3.5%***		
***fully disagree***	5.3% (*n* = 809)	***6.6%*** (*n* = 227)	6.3% (*n =* 269)	***3.5%*** (*n* = 313)		
***I get a referral from my GP to see a medical specialist.***					***0.006***	***0.012***
***fully agree***	67.0%	***59.7%***	***65.4%***	***73.6%***		
***mostly agree***	16.4%	***20.2%***	***15.2%***	***14.7%***		
***not sure***	2.8%	***4.0%***	***1.5%***	***3.2%***		
***mostly disagree***	6.8%	***9.7%***	***7.8%***	***3.8%***		
***fully disagree***	7.0% (*n* = 811)	***6.6%*** (*n =* 228)	***10.0%*** (*n =* 269)	***4.8%*** (*n =* 314)		
I discuss the results of medical specialist consultations with my GP.					0.276	0.953
fully agree	61.1%	56.1%	62.1%	63.9%		
mostly agree	19.9%	22.4%	20.1%	17.9%		
not sure	3.5%	4.8%	3.0%	2.9%		
mostly disagree	7.0%	6.6%	6.7%	7.7%		
fully disagree	8.5% (*n* = 810)	10.1% (*n =* 228)	8.2% (*n =* 269)	7.7% (*n =* 313)		
I trust my GP more than any other physician.					0.737	0.438
fully agree	54.4%	59.2%	50.1%	54.6%		
mostly agree	17.9%	16.2%	20.5%	16.9%		
not sure	11.7%	8.8%	14.5%	11.5%		
mostly disagree	4.7%	3.5%	5.6%	4.8%		
fully disagree	11.2% (*n =* 810)	12.3% (*n =* 228)	9.3% (*n =* 269)	12.1% (*n =* 313)		
My GP provides excellent information on my treatment.					0.704	0.170
fully agree	80.4%	82.5%	78.8%	80.3%		
mostly agree	13.8%	12.7%	14.9%	13.7%		
not sure	3.6%	3.1%	5.2%	2.6%		
mostly disagree	1.4%	1.3%	0.7%	1.9%		
fully disagree	0.9% (*n =* 811)	0.4% (*n =* 228)	0.4% (*n =* 269)	1.6% (*n* = 314)		

u/r: comparison “urban areas” vs. “rural areas”; e/r: comparison “environs” vs. “rural areas”

Statistically significant results (p ≤ 0.05) are shown in bolt and italic

The total score of commitment to the GP was in the arithmetic average of 20 points and in the median of 22 of 24 possible points. 51.2% of the patients had a commitment to their GP ranging between the median and maximum (“strong commitment to the GP”). 48.8% of the patients had a commitment to their GP below the median (“low commitment to the GP”). Comparisons of these two groups can be found in Tables 1 and 2. Patients with a stronger commitment to their GP were older and more likely male. They usually stated a lower income and more often had a lower and less frequently a higher education. In addition and more frequently, they had already been retired, were less likely to be an employee and were more often registered job-seekers.

Furthermore, patients with a stronger commitment to their GP had a higher number of chronic medical conditions. This involved in particular high blood pressure, diabetes mellitus, CHD, chronic fatigue, atherosclerosis/PAOD and cardiac valve disorders. Patients with a stronger commitment to their GP had also more contact with their GP and less contact with medical specialists than patients with a lower commitment to their GP.

### Psychometric construct validation

In the GP population described here, the six items with Cronbach’s α = 0.735 showed an adequate high internal consistency. The explorative factor analysis showed with one single extracted factor (eigenvalue = 2.178) the one-dimensional property of the construct. At the same time, items 1 to 4 consistently showed a higher factor loading than items 5 and 6 (see Table 4). The item-total correlation could always be considered satisfactory with items 1 to 4 also consistently displaying a higher correlation coefficient than items 5 to 6.

**Table 4:** Psychometric construct validation “commitment to GP” from F-HaBi

	Factor loading to extracted factor*	Item-total correlation**
Item 1: When I have health problems, I consult my GP first.	0.626	0.529
Item 2: When I think that I have to see a medical specialist, I consult my GP in advance.	0.815	0.662
Item 3: I get a referral from my GP to see a medical specialist.	0.734	0.578
Item 4: I discuss the results of medical specialist consultations with my GP.	0.587	0.533
Item 5: I trust my GP more than any other physician.	0.341	0.311
Item 6: My GP provides excellent information on my treatment.	0.351	0.340

* Results of an exploratory factor analysis

** Results of Part-Whole-corrected Pearson correlations between items und summary scores

### Predictors of commitment to GP

The correlation between region, sociodemography, healthcare utilisation and patients’ commitment to their GP is illustrated in Table 5. Regarding the multivariate multilevel analysis, commitment to the GP in lower urban density was more pronounced in men and in cases of higher numbers of chronic medical conditions. This model did not verify a correlation with the patients’ ages. This changed after including healthcare utilisation which, aside from identifying advanced age as predictor of a stronger commitment to the GP, also resulted in a significant improvement of the model fit (*p* = 0.002). Additionally, the patient’s commitment to his GP was even stronger, the more contact he had with his GP and the less contact he had with medical specialists. In the end, the inclusion of income, education, and professional situation resulted in an additional significant improvement of the model fit (*p* < 0.001). However, age lost again its statistical significance in this model. On the other hand, tertiary education and a higher income were associated with a less pronounced commitment to the GP. The professional situation had no effect on the commitment to the GP in this model.

**Table 5:** Correlations between regions, sociodemography, healthcare utilisation and commitment to GP: results of a multivariate linear regression adjusted for random effects on the levels of German federal states, administrative districts and GP practices (*n* = 753)

	Model 1		Model 2		Model 3	
	ß (95% CI)	p	ß (95% CI)	p	ß (95% CI)	p
***Region***						
***urban areas*vs.*rural areas***	***−1.06 (−1.81/−0.31)***	***0.006***	***−1.05 (−1.79/−0.31)***	***0.005***	***−0.85 (−1.58/−0.12)***	***0.022***
***environs*vs.*rural areas***	***−0.90 (− 1.74/−0.07)***	***0.034***	***−0.96 (− 1.79/− 0.13)***	***0.024***	***−0.80 (− 1.57/− 0.02)***	***0.045***
***Age (per 10 years)***	0.20 (− 0.04/0.43)	0.098	***0.28 (0.05/0.51)***	***0.018***	0.25 (− 0.07/0.58)	0.126
***Sex: male*vs.*female***	***1.09 (0.48/1.70)***	***< 0.001***	***1.02 (0.42/1.62)***	***0.001***	***1.14 (0.53/1.74)***	***< 0.001***
***Number of medical chronic conditions***	***0.17 (0.08/0.27)***	***< 0.001***	***0.15 (0.05/0.25)***	***0.003***	***0.10 (0.00/0.20)***	***0.043***
***Contacts with GP***			***0.33 (0.17/0.49)***	***< 0.001***	***0.30 (0.15/0.46)***	***< 0.001***
***Contacts with medical specialists***			***−0.86 (−1.49/−0.23)***	***0.007***	***− 0.75 (− 1.36/− 0.13)***	***0.018***
***Education (pursuant to CASMIN):***						
medium vs. low					−0.60 (−1.29/0.08)	0.086
***high*vs.*low***					***−1.74 (−2.68/−0.81)***	***< 0.001***
***Equivalised disposable income: natural logarithm***					***−0.93 (−1.56/− 0.30)***	***0.004***
Professional situation						
employed					0.24 (−1.04/1.52)	0.714
self-employed/freelancer					−0.57 (− 2.21/1.08)	0.499
housewife/homemaker					0.88 (−1.11/2.88)	0.385
job-seeking/unemployed					0.73 (−1.39/2.85)	0.499
retiree/pensioner					0.01 (−1.45/1.46)	0.993

Statistically significant results (p ≤ 0.05) are shown in bolt and italic

## Discussion

### Main findings

Commitment to the GP is a healthcare epidemiological construct for describing a patient’s willingness to use the GP as coordinator of his medical treatment. The psychometric validation of a population of GP patients verified the commitment to the GP to be a one-dimensional construct with adequate high internal consistency und satisfactory item-total correlation of all items.

On average, the patients of the present investigation, who had all been recruited from systematically selected regions of northern Germany, attained relatively high scores regarding the total scores of commitment to their GP. However, there were clear differences according to sex, morbidity, education, income and degree of their residency’s urban density. In fact, although age was identified to be a predictor for commitment to the GP, it lost, however, depending on the constellation of the model’s enclosed covariates, its statistical significance. Additionally, there was a correlation between the commitment to the GP and contacts to GPs and medical specialists. Due to the cross-sectional design of the study, it could not be clarified whether the commitment to the GP depended on healthcare utilisation or healthcare utilisation on the commitment to the GP. Thus, advocating the working hypothesis that the correlation is a two-way flow.

### Strengths and limitations of the survey

One of our study’s strengths is the fact that GP practices had been included via a quota sampling into the study. As a result, 91.9% of the administrative districts in the survey area could be illustrated and GPs of less favoured areas, such as Kalbe (Milde) or Helgoland, which are difficult to reach by public transport, could also be included into the study. However, in the end, we were able to interview only 25.1% of the patients contacted to participate in the study which might result in a limited representativeness of our patient sample.

The representativeness of our sample might also be affected by our eligibility criteria and the recruitment procedure. All participants of our study were exclusively from the regions of northern Germany so that our sample might possibly not represent the rest of Germany. The interviewed patients had been recruited through GP practices and our inclusion criteria selected patients who have been registered with their GP for at least 3 years and had seen their GP within the last 3 months. For that reason, the patients from our study probably had a higher commitment to their GP than the general population. It should also be noted that patients with relevant functional limitations regarding participation in the study, patients lacking German language skills and patients with missing capacity to consent could not be interviewed and that these patient groups were thus also not represented in the results presented here.

There were no statistically significant differences regarding regional category, age, sex, workload and the total number of treated patients between GPs who recruited patients for our survey and GPs who failed to do so. We were unable to do a sample size calculation because this was an observational study with multiple outcomes. Therefore, we might have missed some differences between the regions due to limited statistical power. Despite the fact that the interviewers had received substantial training and had been supervised in regular meetings throughout the entire survey period, the patients’ answers might – as in any other survey-based study, too, have been influenced by memory gaps, errors or social desirability. However, additional strengths worth mentioning are the applied statistical methods that also adequately facilitated taking potential confounders and the cluster structure of the dataset into account.

### Comparison with literature and discussion of results

In a Forsa survey commissioned by the German association of national health insurances, 96% of 1000 participants stated to be very content or content with the healthcare provided by their GP [17]. A study by Detollenaere et al. exploring social differences in patient satisfaction of their GP within 31 European countries reported similar satisfaction rates [18]. The generally very high patient satisfaction with their GP in our survey might also be mirrored by the overall relatively strong commitment of the respective patients to their GP.

Compared to patients from rural areas, patients from urban areas clearly had a less pronounced commitment to their GP – predominantly regarding attaining and preliminary discussion for a referral. This finding might be explained, at least to a certain extent, by the regionally different number of medical specialists. A study conducted by the Robert-Koch-Institute, for example, showed that the density of psychotherapists varied 76-fold and the density of neurologists 17-fold between the individual regions of Germany [19]. GPs and patients realise that rural regions in particular are suffering from a undersupply of medical specialists [20]. Thus, 33% of the surveyed patients from rural areas in the already mentioned Forsa study were very discontent with medical specialists’ appointment allocations compared to 18% of patients surveyed in urban areas [17]. Other studies show similar results [21].

A study about predictors of having no GP by Tillmann et al. showed that the odds of having no GP significantly decreased with age and the presence of chronic conditions and increased for living in urban areas [22]. Vice versa, our study revealed higher commitment to the GP for patients from rural areas. Investigations of the HzV in Germany by Schnitzer et al. and Kürschner et al. report that older patients or retirees participate more often in GP models [10, 11]. The age effect could not be clearly determined by our study because, depending on the statistical model, there was at times a correlation and then again no correlation with the commitment to the GP. The presence of medically diagnosed conditions and belonging to the social middle and lower class are linked to more willingness to participate in HzV [10]. The results of our investigation imply similar correlations with the commitment to the GP. Patients with lifestyle-associated medical conditions, such as cardiovascular conditions or diabetes, which occur more often in patients of lower socioeconomic status [23, 24], are particularly dependent on continuous (primary) medical care. Thus, the stronger commitment of these patient groups to their GPs is good news.

The European Social Survey (2014) show that higher educated participants were more likely to use health care specialists in 11 countries (UK, Sweden, Austria, Norway, Finland, the Czech Republic, France, Germany, Spain, Poland and Portugal) [25]. Schnitzer et al. report of a negative effect of tertiary education and urban residency in participating in HzV claiming that GP models can offset the more frequent utilisation of medical specialists by patients with a higher formal education in large cities [11]. Our analyses show a negative correlation between commitment to the GP and number of contacts with medical specialists. This implies that a likely increase in the commitment to the GP, eg, by participating in HzV, may indeed have an effect on consulting medical specialists.

### Implications for research and clinical practice

Considering age, sex, region of residency, morbidity and socioeconomic status, patients who showed a high degree of willingness to use their GP as coordinator of their treatment made somewhat more frequent use of their GP but considerably less use of medical specialists than patients with a low commitment to their GP. Bearing in mind that overuse and/or misuse is all the more likely the more therapists a patient is consulting [26], using the coordinating function of the GP – as conveyed in the future positions (“*Zukunftspositionen*”) of the German Association of General Medicine and Family Medicine (DEGAM) – might thus protect patients against “too much and the wrong medicine” [27].

Patients from regions with a higher urban density showed a lower commitment to their GP than patients from less-favoured regions. One explanation might be that urban patients are used to directly consulting medical specialists due to the large number of available medical specialists and the comparably short waiting times for appointments and therefore resort less to GP coordination.

Likewise, the lower commitment of women to their GP may be explained by the fact that they are often regularly cared for by gynaecologists and thus do not experience the GP as a central figure for coordinating the treatment. Additionally, the treatment coordination by a GP does not seem to be as appealing to those with a higher education and higher income and is therefore used less than by people with less income and a more remote education background.

## Conclusions

On average, the patients’ commitment to their GP was relatively strong, but there were large differences between patient groups. HzV is an already existing and tested intervention for increasing the commitment to the GP [10, 11]. Since, however, particularly the groups with the least commitment to a GP also have the lowest willingness to participate in HzV, HzV in its present form seems to be least suited to appeal to the aforementioned patient groups.

A potential first starting point for increasing the commitment to a GP might be to provide the identified patient groups with specific information on the importance of GP coordination. In addition, qualitative studies with GPs and patients should explore how GP medical care can be made more appealing to patient groups like women or patients from regions of higher urban density, thus facilitating designing special GP models specifically focusing on groups with the lowest commitment to a GP.

## Supplementary information

**Additional file 1: Table A**: planned and completed size in the region “urban areas”. **Table B**: planned and completed sample size in the region “environs”. **Table C**: planned and completed sample size in the region “rural areas”.

**Additional file 2.** German version of the questionnaire on intensity of the commitment to the GP

**Additional file 3.** English version of the questionnaire on intensity of the commitment to the GP

## Data Availability

The datasets analysed during the current study are not publicly available as data sharing with other researchers was not part of patients’ informed consent.

## Abbreviations

CASMIN: Comparative Analysis of Social Mobility in Industrial Nations
CHD: Coronary heart disease
COPD: Chronic obstructive pulmonary disease
DEGAM: German Association of General Medicine and Family Medicine
EDP: Electronic Data Processing
F-HaBi: Questionnaire on intensity of the commitment to the GP (Fragebogen zur Intensität der Hausarztbindung)
GP: General practitioner
HzV: GP-centred healthcare (Hausarztzentrierte Versorgung)
PAOD: peripheral arterial occlusive disease
