# Supplementary material for: Regional and patient-related factors influencing the willingness to use general practitioners as coordinators of the treatment in northern Germany - results of a cross-sectional observational study
Source: BMC Fam Pract. 2020 Jun 17;21:110. doi: 10.1186/s12875-020-01180-3 (PMC7302141; doi:10.1186/s12875-020-01180-3)
Supplement: Supplementary file 1 — Additional file 1: Table A: planned and completed size in the region “urban areas”. Table B: planned and completed sample size in the region “environs”. Table C: planned and completed sample size in the region “rural areas”. [file 12875_2020_1180_MOESM1_ESM.pdf]

**Additional file 1:**

**Table A: planned and completed size in the region „urban areas“**

| **Administration district** | **Inhabitants** | **GPs planned** | **Recruited GPs** | **Contacted patients** | **GPs of participating patients** | **Interviewed patients** |
|---|---|---|---|---|---|---|
| **Bremen** | | | | | | |
| - Bremen Centre | 18.231 | 1 | 2 | 30 | 1 | **5** |
| - Bremen North | 97.460 | 3 | 5 | 75 | 5 | **26** |
| - Bremen East | 223.753 | 6 | 2 | 15 | 1 | **9** |
| - Bremen South | 126.710 | 3 | 2 | 30 | 2 | **9** |
| - Bremen West | 91.310 | 2 | 5 | 60 | 4 | **13** |
| **Bremerhaven** | 114.025 | 3 | 3 | 30 | 2 | **8** |
| **Hamburg** | | | | | | |
| - Hamburg Altona | 267.058 | 7 | 11 | 150 | 8 | **32** |
| - Hamburg Bergedorf | 124.998 | 4 | 4 | 45 | 3 | **12** |
| - Hamburg Eimsbüttel | 258.865 | 7 | 2 | 30 | 2 | **7** |
| - Hamburg Harburg | 160.211 | 4 | 5 | 60 | 3 | **9** |
| - Hamburg Centre | 296.410 | 8 | 10 | 120 | 6 | **13** |
| - Hamburg North | 302.242 | 8 | 10 | 120 | 6 | **34** |
| - Hamburg Wandsbek | 424.146 | 11 | 13 | 150 | 8 | **36** |
| **Kiel** | 246.306 | 7 | 4 | 45 | 2 | **14** |
| **Lübeck** | 216.253 | 6 | 4 | 60 | 1 | **1** |
| | | 80 | 82 | 1.020 | 54 | **228** |

**Table B: planned and completed sample size in the region "environs"**

| Administration district | Inhabitants | GPs planned | Recruited GPs | Contacted patients | GPs of participating patients | Interviewed patients |
|---|---|---|---|---|---|---|
| **Altmarkkreis Salzwedel** | 86.164 | 2 | 4 | 45 | 2 | **11** |
| **Delmenhorst** | 76.323 | 2 | 1 | 0 | 0 | **0** |
| **Diepholz** | 16.692 | 1 | 0 | 0 | 0 | **0** |
| **Harburg** | 248.122 | 7 | 9 | 105 | 7 | **36** |
| **Herzogtum Lauenburg** | 192.999 | 5 | 7 | 45 | 3 | **6** |
| **Neumünster** | 79.197 | 2 | 3 | 45 | 3 | **17** |
| **Nordwestmecklenburg** | 156.270 | 4 | 6 | 75 | 5 | **17** |
| **Oldenburg** | 128.608 | 4 | 2 | 30 | 2 | **9** |
| **Osterholz** | 113.579 | 3 | 0 | 0 | 0 | **0** |
| **Ostholstein** | 199.574 | 5 | 5 | 60 | 3 | **9** |
| **Pinneberg** | 307.471 | 8 | 12 | 135 | 9 | **36** |
| **Plön** | 128.304 | 4 | 4 | 45 | 3 | **10** |
| **Rendsburg-Eckernförde** | 270.378 | 7 | 11 | 150 | 9 | **35** |
| **Schwerin** | 96.800 | 3 | 3 | 45 | 1 | **4** |
| **Segeberg** | 267.503 | 7 | 8 | 105 | 6 | **29** |
| **Stade** | 200.054 | 5 | 13 | 105 | 6 | **29** |
| **Stormarn** | 239.614 | 7 | 8 | 90 | 4 | **13** |
| **Verden** | 134.645 | 4 | 5 | 45 | 3 | **8** |
| | | 80 | 101 | 1.125 | 66 | **269** |

**Table C: planned and completed sample size in the region “rural areas”**

| Administration district* | Inhabitants | GPs planned | Recruited GPs | Contacted patients | GPs of participating patients | Interviewed patients |
|---|---|---|---|---|---|---|
| **Celle** | | | | | | |
| - City of Celle | 69.748 | 2 | 1 | 0 | 0 | **0** |
| - rural area | 108.223 | 4 | 8 | 60 | 4 | **18** |
| **Cuxhaven** | | | | | | |
| - City of Cuxhaven | 48.264 | 2 | 1 | 0 | 0 | **0** |
| - City of Geestland | 30.936 | 1 | 0 | 0 | 0 | **0** |
| - rural area | 118.903 | 4 | 5 | 75 | 5 | **30** |
| **Dithmarschen** | | | | | | |
| - City of Heide | 21.422 | 1 | 0 | 0 | 0 | **0** |
| - rural area | 111.495 | 4 | 10 | 105 | 6 | **31** |
| **Gifhorn** | | | | | | |
| - City of Gifhorn | 41.905 | 1 | 1 | 15 | 1 | **1** |
| - rural area | 132.300 | 5 | 3 | 15 | 1 | **1** |
| **Heidekreis** | | | | | | |
| - City of Soltau | 21.414 | 1 | 1 | 0 | 0 | **0** |
| - City of Walsrode | 23.219 | 1 | 3 | 30 | 2 | **7** |
| - rural area | 95.631 | 3 | 5 | 60 | 4 | **14** |
| **Ludwigslust-Parchim** | 214.113 | 8 | 8 | 75 | 4 | **16** |
| **Lüchow-Dannenberg** | 50.128 | 2 | 9 | 120 | 6 | **13** |
| **Lüneburg** | | | | | | |
| - City of Lüneburg | 74.072 | 3 | 4 | 30 | 2 | **14** |
| - rural area | 106.647 | 4 | 5 | 60 | 4 | **32** |
| **Nienburg** | | | | | | |
| - City of Nienburg | 31.193 | 1 | 2 | 30 | 2 | **8** |
| - rural area | 89.439 | 3 | 2 | 30 | 2 | **10** |
| **North Frisia** | | | | | | |
| - City of Husum | 22.430 | 1 | 1 | 15 | 1 | **4** |
| - rural area | 141.260 | 5 | 7 | 105 | 5 | **26** |
| **Rotenburg (Wümme)** | | | | | | |
| - City of Rotenburg | 21.392 | 1 | 0 | 0 | 0 | **0** |
| - rural area | 141.861 | 5 | 4 | 45 | 3 | **26** |
| **Schleswig-Flensburg** | | | | | | |
| - City of Schleswig | 24.266 | 1 | 0 | 0 | 0 | **0** |
| - rural area | 172.573 | 6 | 4 | 45 | 3 | **18** |
| **Steinburg** | | | | | | |
| - City of Itzehoe | 31.771 | 1 | 2 | 30 | 2 | **5** |
| - rural area | 99.686 | 4 | 4 | 45 | 3 | **13** |
| **Uelzen** | | | | | | |
| - City of Uelzen | 33.782 | 1 | 0 | 0 | 0 | **0** |
| - rural area | 59.349 | 2 | 5 | 60 | 4 | **21** |
| **Wesermarsch** | | | | | | |
| - City of Nordenham | 26.325 | 1 | 0 | 0 | 0 | **0** |
| - rural area | 62.914 | 2 | 2 | 30 | 2 | **6** |
| | | 80 | 97 | 1.080 | 66 | **314** |

* stratified by cities of more than 20,000 inhabitants and rural areas.
