# Supplementary material for: Regional and patient-related factors influencing the willingness to use general practitioners as coordinators of the treatment in northern Germany - results of a cross-sectional observational study
Source: BMC Fam Pract. 2020 Jun 17;21:110. doi: 10.1186/s12875-020-01180-3 (PMC7302141; doi:10.1186/s12875-020-01180-3)
Supplement: Supplementary file 2 — Additional file 2. German version of the questionnaire on intensity of the commitment to the GP [file 12875_2020_1180_MOESM2_ESM.pdf]

Additional file 2: German version of the questionnaire on intensity of the commitment to the GP

Fragebogen zur Intensität der Hausarztbindung (F-HaBi)

Als nächstes würden wir von Ihnen gerne einige Informationen zu Ihrer hausärztlichen Versorgung haben.

F-HaBi1 **Haben Sie einen Hausarzt/eine Hausärztin?**
☐ ja
☐ nein

F-HaBi2 **Wenn ja, wie häufig suchen Sie Ihren Hausarzt/Ihre Hausärztin pro Jahr auf?** *Kommentar: gemeint sind alle Besuche mit direkten Arztkontakt (nicht nur Rezept abholen) sowie Hausbesuche*
⌊ ⌋⌊ ⌋ mal

**Inwieweit trifft jede der folgenden Aussagen auf Sie zu?**		**trifft ganz zu**	**trifft weit-gehend zu**	**weiß nicht**	**trifft weit-gehend nicht zu**	**trifft über haupt nicht zu**
F-HaBi3	**Wenn ich ein Gesundheitsproblem habe, suche ich zuerst meinen Hausarzt/meine Hausärztin auf.**	☐	☐	☐	☐	☐
F-HaBi4	**Wenn ich denke, dass ich zu einem Facharzt/Spezialisten muss, bespreche ich das vorab mit meinem Hausarzt/meiner Hausärztin.**	☐	☐	☐	☐	☐
F-HaBi5	**Ich hole mir von meinem Hausarzt/meiner Hausärztin eine Überweisung, um zum Facharzt/Spezialisten zu gehen.**	☐	☐	☐	☐	☐
F-HaBi6	**Ich bespreche mit meinem Hausarzt/meiner Hausärztin die Ergebnisse von Terminen bei anderen Fachärzten/Spezialisten.**	☐	☐	☐	☐	☐
F-HaBi7	**Ich vertraue meinem Hausarzt/meiner Hausärztin mehr als anderen Ärzten.**	☐	☐	☐	☐	☐
F-HaBi8	**Mein Hausarzt/meine Hausärztin gibt mir sehr gute Informationen über meine Behandlung.**	☐	☐	☐	☐	☐
