# Supplementary material for: Regional and patient-related factors influencing the willingness to use general practitioners as coordinators of the treatment in northern Germany - results of a cross-sectional observational study
Source: BMC Fam Pract. 2020 Jun 17;21:110. doi: 10.1186/s12875-020-01180-3 (PMC7302141; doi:10.1186/s12875-020-01180-3)
Supplement: Supplementary file 3 — Additional file 3. English version of the questionnaire on intensity of the commitment to the GP [file 12875_2020_1180_MOESM3_ESM.pdf]

Additional file 3: English version of the questionnaire on intensity of the commitment to the GP

Now we would like to get some information about your primary care.

F-HaBi1 **Do you have a general practitioner?**
☐ yes
☐ no

F-HaBi2 **If so, how often do you see your general practitioner each year?** *Comment: This includes all consultations at the practice with personal contact to the GP and home visits. It does not include visits at the practice for picking up prescriptions without personal contact to the GP.*
└ ┘└ ┘ times

To what extent do the following statements apply to you?		fully agree	mostly agree	not sure	mostly dis-agree	fully dis-agree
F-HaBi3	**When I have health problems, I visit my GP first.**	☐	☐	☐	☐	☐
F-HaBi4	**When I think that I have to see a medical specialist, I consult my GP in advance.**	☐	☐	☐	☐	☐
F-HaBi5	**I get a referral from my GP to see a medical specialist.**	☐	☐	☐	☐	☐
F-HaBi6	**I discuss the results of medical specialist consultations with my GP.**	☐	☐	☐	☐	☐
F-HaBi7	**I trust my GP more than any other physician.**	☐	☐	☐	☐	☐
F-HaBi8	**My GP provides excellent information on my treatment.**	☐	☐	☐	☐	☐
